# Consensus Statement on Public Involvement and Engagement with Data Intensive Health Research

**DOI:** 10.23889/ijpds.v4i1.586

**Published:** 2019-02-12

**Authors:** Mhairi Aitken, Mary P Tully, Carol Porteous, Simon Denegri, Sarah Cunningham-Burley, Natalie Banner, Corri Black, Michael Burgess, Lynsey Cross, Johannes JM van Delden, Elizabeth Ford, Sarah Fox, Natalie K Fitzpatrick, Kay Gallacher, Catharine Goddard, Lamiece Hassan, Ron Jamieson, Kerina H Jones, Minna Kaarakainen, Fiona Lugg-Widger, Kimberlyn McGrail, Anne McKenzie, Rosalyn Moran, Madeleine J Murtagh, Malcolm Oswald, Alison Paprica, Nicola Perrin, Emma Victoria Richards, John Rouse, Joanne Webb, Donald J Willison

**Affiliations:** 1 University of Edinburgh, Usher Institute of Population Health Sciences and Informatics, NINE Edinburgh BioQuarter, 9 Little France Road, Edinburgh, EH16 4UX; 2 University of Manchester, Div Pharmacy and Optometry, Oxford Road, Manchester, M13 9PL, UK; 3 NIHR, INVOLVE, Alpha House, University of Southampton Science Park, Chilworth, Southampton, SO16 7NS, UK; 4 Wellcome Trust, Gibbs Building, 215 Euston Road, London NW1 2BE, UK; 5 University of Aberdeen, Institute of Applied Health Sciences, Aberdeen Centre for Health Data Science, School of Medicine, Medical Science and Nutrition, Foresterhill, Aberdeen AB25 2ZD; 6 University of British Columbia, Faculty of Medicine, School of Population and Public Health, 2206 East Mall Vancouver, BC Canada V6T 1Z3; 7 Swansea University. Population Data Science, Medical School, Singleton Campus, Swansea SA2 8PP, UK; 8 University Medical Centre Utrecht, Julius Centre for Health Sciences, Uniwersiteitsweg 100, 3584 CG Utrecht, Netherlands; 9 Brighton and Sussex Medical School, Watson Building, University of Brighton, Falmer, Brighton, BN1 9PH, UK; 10 UCL Institute of Health Informatics, 222 Euston Road, London, NW1 2DA, UK; 11 HeRC Patient & Public Involvement (H@PPI) Forum, The Health eResearch Centre Vaughan House Portsmouth Street Manchester M13 9GB; 12 University of Dundee, School of Life Sciences, University of Dundee, Dow Street, Dundee, DD1 5EH, UK; 13 Public Panel, Farr Institute of Health Informatics Research, Scotland, UK; 14 University of Helsinki, Center for Consumer Society Research, PL 24 (Unioninkatu 40) HELSINGIN YLIOPISTO Finland; 15 Cardiff University, Centre for Trials, 702C, Neuadd Meirionnydd, Heath Park, Cardiff, UK; 16 University of Western Australia, Faculty of Health and Medical Sciences, School of Population and Global Health, 35 Stirling Highway, 6009 Perth Australia; 17 EKOS- Social and Environmental Research Associates; 18 Newcastle University, The School of Geography, Politics and Sociology, Windsor Terrace, Newcastle upon Tyne NE2 4HE , UK; 19 University of Toronto, Dalla Lana School of Public Health, Institute of Health Policy, Management and Evaluation, Health Sciences Building,155 College Street, Toronto, ON M5T 3M6, Canada; 20 Understanding Patient Data, Wellcome Trust; 21 CIPHER Consumer Panel; 22 Public Panel, Farr Institute of Health Informatics Research, London, UK; 23 Administrative Data Service (Administrative Data Research Network), University of Essex, Wivenhoe Park, Colchester, CO4 3SQ, UK

## Introduction

This consensus statement reflects the deliberations of an international group of stakeholders with a range of expertise in public involvement and engagement (PI&E) relating to data-intensive health research. It sets out eight key principles to establish a secure role for PI&E in and with the research community internationally and ensure best practice in its execution. Our aim is to promote culture change and societal benefits through ensuring a socially responsible trajectory for innovations in this field.

**Our key premise is that the public should not be characterised as a problem to be overcome but a key part of the solution to establish socially beneficial data-intensive health research for all.**

### Data-intensive health research

Data-intensive health research is a fast moving field as ever more data are generated, new computational abilities are developed, a wider variety of data and data types are linked, and novel applications in health care are fostered. (Box 1)

Box 1. What is Data-Intensive Health Research? The term data-intensive health research refers to forms of health research which are conducted through the linkage and analysis of data. These data can take many forms and be derived from diverse sources.

Some examples of the types of data which are used in this research include:

- Data from patient records;
- Administrative data (e.g. from social care, housing or education);
- Data from registries;
- Genomic data (e.g. from biobanks);
- Data generated through use of apps;
- Social media data.

Research may use data from a single source or link data from multiple sources together. These data are de-identified.

This research is conducted for a range of purposes including:

- Clinical decision support;
- Monitoring drug safety;
- Developing predictive models;
- Examining connections between social or behavioural factors and health outcomes;
- Audit of services.

Internationally, the significant interest in research uses of routinely collected health-relevant data has led to the creation of data infrastructures in different jurisdictions which facilitate and undertake data-intensive health research including Health Data Research UK (HDR UK), in the U.K., the Western Australia Data Linkage System and Population Health Research Network in Australia, and Population Data BC in Canada.

These developments have led to calls for a new social contract between health related science and the public [1,2] and the relationship between science and society has come under increasing scrutiny. Recent highly publicised controversies over data use in research, such as with national data records systems in England [2] and Australia [3] suggest that public acceptance cannot be assumed. Yet, public support is needed to foster innovation and research: such research needs to reflect public values and interests if it is to gain such support. Publics1The plural ‘publics’ is used to reflect the heterogeneity of the different groups within the general public. then hold a key position as both generators and recipients of the potential benefits of health related data science.

There is now broad recognition of the value of public knowledge and expertise in relation to science, technology and medicine [4, 5] and PI&E is increasingly part of the contemporary landscape of health research and health care innovation [6, 7]. However, while scientists, funders and policy-makers increasingly emphasise the importance of dialogue, their efforts have often been criticised for being tokenistic or cosmetic [8, 9]. Although some consensus is emerging around the importance of PI&E, commitments and practices are varied. The emergence of data intensive research, and the social contract upon which it relies, demands that we move beyond rhetorical commitments and engage anew with clearly stated principles to build PI&E into data-intensive health research at all levels. Renewal is required if data-intensive health research is to develop in socially acceptable and ethically robust ways [10-13].

### Characterising public involvement and engagement

PI&E is an important mechanism for engendering and sustaining the necessary social licence for data-intensive health research, and members of the public have diverse perspectives and insights which can and should play an important role in shaping research infrastructures, processes and applications. The processes and purposes of PI&E are also diverse. These range from raising awareness of current research, consulting members of the public on their views about health research, working in partnership, to empowering members of the public to play a role in shaping current or future research or governance practices [12]. Some examples of the range of approaches which can be used in PI&E are summarised in Box 2.

PI&E relating to data-intensive health research takes place at many different scales, including:

- Wide-scale public conversations about uses or potential uses of data in health research;
- PI&E to inform or co-design the development of policies or governance practices relating to uses of data in health research;
- Engagement or involvement of members of the public in governance decisions about data access and use;
- Engagement or involvement of members of the public at different phases in particular research projects;
- Analysing and disseminating the results of research using data in ways which will support improvements in health care and systems.

Box 1. What is Data-Intensive Health Research? Public Involvement and Engagement (PI&E) can serve a variety of purposes and take diverse approaches. These approaches can be categorised as: Awareness Raising, Consultation and Empowerment and, while not mutually exclusive, lead to a variety of different methods being used. Awareness Raising involves information being disseminated to the public. Through consultation information (including ideas, preferences or concerns) is sought from the public. Empowerment can occur through developing skills, capacities and social capital and enhancing democracy [12].

Some examples of methods which have been used in PI&E relating to data-intensive health research are shown here:

**Figure 1: Adapted from Davidson et al 2013 [12]:**
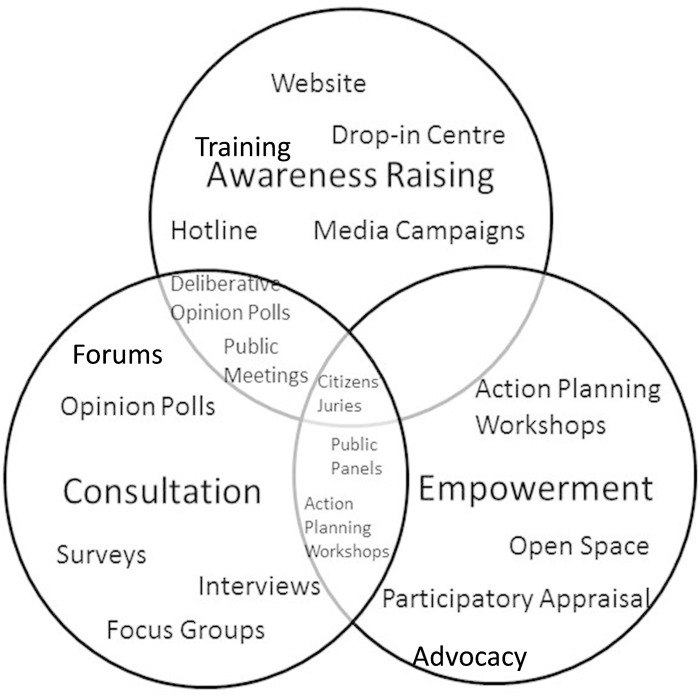

While the methods used may not vary greatly from PI&E with other types of research, or other policy areas, there are particular features of data-intensive health research which make PI&E in this area worthy of special consideration. These are addressed in detail below.

### Developing the consensus statement

A workshop was held in Manchester, UK in April 2017. This was organised by the Public Engagement research team of the Farr Institute of Health Informatics Research (www.farrinstitute.org), and chaired by Simon Denegri from INVOLVE. The workshop was attended by 31 international participants, identified from literature searches, networks and recommendations. They represented a range of practical experience of PI&E and/or research into public attitudes around data use/linkage. Consensus workshop participants included practitioners, policy stakeholders and academics (principally social scientists). In addition, representatives of each of the four Farr Institute Public Panels also participated. Workshop participants came from the U.K., Ireland, Australia, Canada, Finland and the Netherlands.

The format comprised plenary and small group discussions when participants moved between four tables, each facilitated by a member of the Farr Institute Public Engagement research team. Key points were summarised on flipcharts and post-it notes. Each of the small group discussions were recorded and transcribed by members of the research team and detailed notes were taken of plenary discussions. A short report of the day was circulated to all participants. A small team (MA; MT; CP; SCB) from the Farr Institute drafted an initial consensus statement based on the day’s discussions and conclusions. This was circulated for comment by the workshop attendees, a small number of invitees who had not been able to attend the workshop and the four Farr Institute Public Panels. The statement went through further iterations in response to comments. All authors have agreed this final version.

The following section sets out why we consider PI&E to require special consideration in relation to data-intensive health research. The statement then delineates key principles that should underpin PI&E in data-intensive health research and drive its implementation.

## Public Involvement and Engagement in Data-Intensive Health Research Requires Special Consideration

Data-intensive health research utilises data from large numbers of people, including whole populations. This presents challenges about whom to engage and whether, or how, PI&E should reach everyone whose data are used. Many other forms of health-related research involve active research participation from patients or publics, and often with smaller overall numbers: in these instances PI&E can be more easily targeted at relevant patient or service user groups.

- **Given the scale of data-intensive health research, involving data from large numbers of people and populations, PI&E is particularly important for awareness raising and for enabling people to participate in processes of research and governance.**

In contrast with much other research, data scientists typically have no contact with those from whom data are derived, creating distance between ‘science’ and ‘society’. Particular effort is required to overcome this and for scientists to develop an understanding of the context and implications of their research.

- **PI&E is particularly important to bridge the gap between data scientists and the people to whom the data relates.**

The data that are used in research relate to people’s lives, yet members of the public may feel disconnected from such information about them as they cannot usually see it, do not know what it relates to, where it is or how it is used. PI&E is a means for increasing public awareness of the data that are collected, generating new forms of data (e.g. through citizen science approaches), and engagement in the ways they are used. This is important for ensuring transparency and also for empowering members of the public to be involved in decisions about how their data are used and/or shared.

- **As well as bridging a gap between researchers and the people to whom the data relates, PI&E can also be a mechanism for bridging a gap between members of the public and the data about them.**

What is legally permissible is not necessarily the same as what is publicly acceptable. As such PI&E has a role to play in ensuring that current and future practices reflect public values and interests, shaping governance in a dynamic and dialogic way.

- **Given that data are often used and linked without individuals’ explicit consent, PI&E has an important role to play in establishing a social licence for current and future practices.**

In many other forms of health-related research, PI&E is essential in order to support specific research, for example as a route to improving recruitment or ensuring consent is informed through accessible materials. In data-intensive health research, the rationale for PI&E is itself at a different scale, dealing with wider social relations and key concepts such as trustworthiness, transparency, fairness and empowerment.

- **Data-intensive health research can promote and enact PI&E to address wider science society relations.**

## Key Principles for Public Involvement and Engagement in Data-Intensive Health Research

This statement is based on the understanding that the public should not be characterised as a problem to be overcome but a key part of the solution to establish socially beneficial data-intensive health research for all. PI&E has a crucial role to play in the fast-moving field of data-intensive health research. To maximise its value and impact it is important to continually improve the scholarship and practice of PI&E in ways that are innovative, inclusive and help secure a sustainable social licence or contract. To do so, this consensus statement posits the following key principles which serve to underpin best practice:

**PI&E relating to data-intensive health research should:**

***1. Have institutional buy-in***

In order to achieve meaningful impact there needs to be a commitment to promote PI&E and to act on the findings. Institutions need to go beyond rhetorical commitments and gestures; this leads to a disconnected PI&E and a failure to close the feedback loop. Given the many different scales at which PI&E takes place and the range of impacts it can bring, institutional buy-in has to occur at all levels and across many sectors, including universities, research institutes, funding bodies and governmental organisations. It is crucial that leadership and governance fully embrace PI&E, recognising the value that it brings and putting in place systems and processes to enable PI&E to be impactful and meaningful. Achieving best practice in PI&E therefore also requires institutional support to develop strategy and skills amongst the research community.

***2. Have clarity of purpose***

The reasons for undertaking PI&E and the ways in which this can effect change should be clearly articulated. This is relevant for an overall PI&E strategy for an organisation, for a programme of research or for an individual researcher or research project. Given the diversity of purposes that PI&E can serve, explicit statements about purpose are required and these should be updated during the process of engagement if necessary. Clarity of purpose is essential in order to set apposite and realistic expectations and select appropriate approaches or evaluation methods.

***3. Be transparent***

PI&E should be conducted with a high degree of transparency both about the data-intensive health research under consideration as well as about its own purpose and processes. This requires openness and accountability to the public about what is happening to data about them, how decisions are made and the ways they can become involved.

***4. Involve two-way communication***

Two-way conversations enable members of the public to be active participants and contributors or partners in involvement and engagement in research related processes. This is a key element that differentiates public engagement from public facing communication strategies. Although the provision of information has an important role in engagement, as awareness raising and in the context of deliberation and dialogue, informing alone should not be characterised as PI&E. Diversity and disagreement are positive features essential for constructive and uncensored dialogue. Consensus is not necessarily a key outcome.

***5. Be inclusive and accessible to broad publics***

Members of the public are competent deliberators who possess valuable expertise and insights relevant to data-intensive health research and its governance. Incorporating public perspectives adds real value and can substantially improve research and governance processes. However, facilitators need to adapt their language and the format of events to ensure accessibility. Those involved in PI&E activities should be supported to make the most of the engagement opportunities offered and to freely and fully articulate their views. PI&E should facilitate the participation of diverse groups and interests.

***6. Be ongoing***

PI&E should form part of an ongoing strategy rather than relying on one-off events. It should start early on in the development of research, governance and other relevant activities. The fast paced field of data-intensive health research requires PI&E to be adaptive to reflect and respond to changing contexts and practices. PI&E should also lead the way in informing research and governance practices as they evolve, in order to ensure these are guided by public values and interests.

***7. Be designed to produce impact***

A success criterion of PI&E has to be identifiable impacts or demonstrable change related to the overall purposes of the engagement. PI&E should therefore be designed with consideration of the potential mechanisms needed to enable change and realise impact with appropriate feedback to participants and wider publics. Change can occur in relation to the direction or focus of PI&E as well as in relation to the project, programme or policy to which it relates. PI&E should lead to positive impacts not just for researchers, institutions or particular projects but also for public participants and wider society.

***8. Be evaluated***

PI&E with data-intensive health research is emerging as an important area within the broader field of PI&E with health care and with science. This field is continually evolving through the development and evaluation of new approaches and methods. Evaluation and critical reflexivity of practitioners are crucial to establishing good practice. Evaluation should relate both to processes and outcomes and engage with different perspectives, including those of public participants. This is key to ensuring the success of particular approaches as well as being vital to the continued development of the science of PI&E itself.

## Conclusion

We propose that these principles should guide approaches to PI&E with data-intensive health research. They should inform the design, implementation and evaluation of PI&E strategies and activities 2Generic PI&E guidance can be found in a range of existing toolkits and resources, including for example:*From the U.K.:*Centre for Public Engagement (NCCPE)’s website contains a range of resources which are helpful for planning, conducting and evaluating public engagement activities: https://www.publicengagement.ac.uk/The National Institute for Health Research (NIHR)’s INVOLVE website contains advice on involving members of the public in research: http://www.invo.org.uk/Sciencewise Public Views Toolkit: http://www.sciencewise-erc.org.uk/cms/public-views-toolkit/*From Ireland:*The Health Research Board (HRB) website offers guidance on public and patient involvement in research: http://www.hrb.ie/funding/funding-schemes/public-and-patient-involvement-in-research/*From Canada:*Canada’s Strategy for Patient-Oriented Research: Patient Engagement Framework at a Glance: http://www.cihr-irsc.gc.ca/e/49232.html*From the Netherlands:*Patientenfederatie Nederland provides useful information and advice: https://www.patientenfederatie.nl/ and shape the mind-sets of researchers, funders and other stakeholders. Adherence to these principles should promote and sustain meaningful PI&E and secure the social licence required to support data-intensive health research now and in the future, with publics as partners not passive recipients of such developments. This will give PI&E authenticity which in turn increases its authority.

This consensus statement is intended to be used in a reflective way to guide practice as the field evolves. Through doing so, this document is intended to remain relevant as the field continues to move in new directions. We invite people to reflect on this when discussing PI&E in data-intensive health research and engage with us to promote best practice locally, nationally and internationally.

## Contribution of Authors

SCB and SD conceived the idea for this project. MA and MPT developed the plans for the consensus workshop. MA, MPT, CP and SCB developed the materials to be used at the consensus workshop, with input from LC, NKF, LH and KHJ. SD chaired and facilitated the consensus workshop. MA, MPT, CP and SCB co-facilitated the consensus workshop. All authors participated in the discussions at the consensus workshop. MA and CP transcribed the consensus workshop discussions. MA synthesised these transcripts to produce an initial report. MA, MPT, CP and SCB drafted the consensus statement. All other authors (SD, NB, CB, MB, LC, JJMD, EF, SF, NKF, KG, CG, LH, RJ, KHJ, MK, FLW, KM, AK, AM, RM, MJM, MO, PAP, NP, ER, JW, DJW) critically revised the consensus statement. The consensus statement developed through iterative drafts in response to contributions from all authors. MA and SCB led in revising the consensus statement in response to authors’ comments. All authors have read and approved the final manuscript.

## References

[1] Academy of Medical Sciences. Exploring A New Social Contract for Medical Innovation. 2015, www.acmedsci.ac.uk

[2] Carter P, Laurie GT, Dixon-Woods M. The social licence for research: why *care.data* ran into trouble. Journal of medical ethics. 23 1 2015:medethics-2014. 10.1136/medethics-2014-102374 PMC4431337 25617016

[3] Garrety K, McLoughlin I, Wilson R, Zelle G, Martin M. National electronic health records and the digital disruption of moral orders. Social Science & Medicine. 1 1 2014;101:70-7. 10.1016/j.socscimed.2013.11.029 24560226

[4] Research Councils UK. Concordat for Engaging the Public with Research. A set of principles drawn up by the Funders of Research in the UK [Online]. 2010. Available: http://www.rcuk.ac.uk/documents/scisoc/concordatforengagingthepublicwithresearch-pdf/ [Accessed 27/06/2017].

[5] Irwin A. The politics of talk: coming to terms with the ‘new’scientific governance. Social studies of science. 4 2006;36(2):299-320. 10.1177/0306312706053350

[6] Domecq JP, Prutsky G, Elraiyah T, Wang Z, Nabhan M, Shippee N, Brito JP, Boehmer K, Hasan R, Firwana B, Erwin P. Patient engagement in research: a systematic review. BMC health services research. 12 2014;14(1):89. 10.1186/1472-6963-14-89 PMC3938901 24568690

[7] Wilson P, Mathie E, Keenan J, McNeilly E, Goodman C, Howe A, Poland F, Staniszewska S, Kendall S, Munday D, Cowe M. ReseArch with Patient and Public invOlvement: a RealisT evaluation–the RAPPORT study. 2015. 10.3310/hsdr03380 26378332

[8] Stilgoe J, Lock SJ, Wilsdon J. Why should we promote public engagement with science?. Public Understanding of Science. 1 2014;23(1):4-15. 10.1177/0963662513518154 PMC5753839 24434705

[9] Kurath M, Gisler P. Informing, involving or engaging? Science communication, in the ages of atom-, bio-and nanotechnology. Public Understanding of Science. 9 2009;18(5):559-73. 20027772 10.1177/0963662509104723

[10] Aitken M, Jorre JD, Pagliari C, Jepson R, Cunningham-Burley S. Public responses to the sharing and linkage of health data for research purposes: a systematic review and thematic synthesis of qualitative studies. BMC medical ethics. 12 2016;17(1):73. 10.1186/s12910-016-0153-x PMC5103425 27832780

[11] Wellcome Trust. The one-way mirror: Public attitudes to commercial access to health data. London: Wellcome Trust. 2016.

[12] Davidson S, McLean C, Treanor S, Aitken M, Cunningham-Burley S, Laurie G, Sethi N, Pagliari C. Public acceptability of data sharing between the public, private and third sectors for research purposes. Scottish Government Social Research; 2013.

[13] Davidson, S., McLean, C., Cunningham-Burley, S. & Pagliari, C. Public Acceptability of Cross-Sectoral Data Linkage (Social Research series) Edinburgh: Scottish Government: 2012

